# Exogenous hydrogen sulfide and methylglyoxal alleviate cadmium-induced oxidative stress in *Salix matsudana* Koidz by regulating glutathione metabolism

**DOI:** 10.1186/s12870-023-04089-y

**Published:** 2023-02-02

**Authors:** Long Guo, Long Ling, Xiaoqian Wang, Ting Cheng, Hongyan Wang, Yanan Ruan

**Affiliations:** School of Life Science, Liaoning University, Shenyang, 110036 China

**Keywords:** Cadmium, *Salix matsudana*, Hydrogen sulfide, Methylglyoxal, Glutathione

## Abstract

**Background:**

Cadmium (Cd) is a highly toxic element for plant growth. In plants, hydrogen sulfide (H_2_S) and methylglyoxal (MG) have emerged as vital signaling molecules that regulate plant growth processes under Cd stress. However, the effects of sodium hydrosulfide (NaHS, a donor of H_2_S) and MG on Cd uptake, physiological responses, and gene expression patterns of Salix to Cd toxicity have been poorly understood. Here, *Salix matsudana* Koidz. seedlings were planted in plastic pot with applications of MG (108 mg kg^− 1^) and NaHS (50 mg kg^− 1^) under Cd (150 mg kg^− 1^) stress.

**Results:**

Cd treatment significantly increased the reactive oxygen species (ROS) levels and malondialdehyde (MDA) content, but decreased the growth parameters in *S. matsudana*. However, NaHS and MG supplementation significantly decreased Cd concentration, ROS levels, and MDA content, and finally enhanced the growth parameters. Cd stress accelerated the activities of antioxidative enzymes and the relative expression levels of stress-related genes, which were further improved by NaHS and MG supplementation. However, the activities of monodehydroascorbate reductase (MDHAR), and dehydroascorbate reductase (DHAR) were sharply decreased under Cd stress. Conversely, NaHS and MG applications restored the MDHAR and DHAR activities compared with Cd-treated seedlings. Furthermore, Cd stress decreased the ratios of GSH/GSSG and AsA/DHA but considerably increased the H_2_S and MG levels and glyoxalase I-II system in *S. matsudana*, while the applications of MG and NaHS restored the redox status of AsA and GSH and further improved glyoxalase II activity. In addition, compared with AsA, GSH showed a more sensitive response to exogenous applications of MG and NaHS and plays more important role in the detoxification of Cd.

**Conclusions:**

The present study illustrated the crucial roles of H_2_S and MG in reducing ROS-mediated oxidative damage to *S. matsudana* and revealed the vital role of GSH metabolism in regulating Cd-induced stress.

**Supplementary Information:**

The online version contains supplementary material available at 10.1186/s12870-023-04089-y.

## Background

Cadmium (Cd) is a nonessential element, and it poses serious danger to plant and animal health due to its mobility and toxicity at relatively low concentrations [1]. Excess amounts of Cd in plants will result in the overproduction of highly reactive oxygen species (ROS), such as singlet oxygen (^1^O_2_), hydrogen peroxide (H_2_O_2_), superoxide radical (O_2_^.-^), and the hydroxyl radical (∙OH) [2], causes oxidative damage to protein and lipids, which further leads to irreversible changes in protein structure and function [3], finally inhibit the photosynthetic process, growth, and yield [4]. In order to minimize Cd-induced oxidative stress, plants have evolved a range of defense systems, including the accumulation of osmolytes and the enhancement of antioxidant enzyme activities, such as superoxide dismutase (SOD), catalase (CAT), and ascorbate peroxidase (APX) [5, 6]. In addition to enzymatic antioxidant systems, antioxidants of non-enzymatic nature, such as glutathione (GSH) and ascorbate (AsA) are also responsible for maintaining the balance between the detoxification and production of ROS [7, 8].

In plants, GSH is a water-soluble non-protein thiol compound and performs a wide range of biochemical functions [9]. Many studies have found that GSH accumulates in response to increased levels of ROS or to compensate for a decrease in the defense capability of other antioxidants [10, 11], and GSH levels are constitutively higher in plants subjected to Cd [12], drought [13], and heat [14] stress conditions. On the one hand, GSH can directly scavenge free radical by reacting with ^1^O_2_, O_2_^.-^, and ∙OH [15]; and on the other hand, GSH is able to induce signal transduction and defense against ROS under stress conditions through regulating the enzymes related to GSH metabolism [10, 15]. Without direct involvement in stress damage alleviation, GSH plays a key role in detoxifying toxic metals by acting as a precursor of phytochelatins (PCs) that bind heavy metals for sequestration in the vacuole [16]. Enhanced levels of the intracellular GSH content and phytochelatin synthase (PCS) activities in *Lotus japonicus* under metal stress indicated that GSH and biosynthesis of PCs participate in sequestration of heavy metals [17].

Methylglyoxal (MG) is a by-product of glycolysis and is cytotoxic to plant cells at high cellular concentrations, but it may act as an important signaling molecule at low concentrations [18]. Previous studies reported that the overaccumulation of MG induced by Cd stress results in severe oxidative damage to plants [19, 20]. Generally, glyoxalase I (Gly I) and glyoxalase II (Gly II) play a pivotal role in MG detoxification by using GSH as a substrate [21]. Recently, the regulation of the glyoxalase I-II system in plants by exogenous application of chemical treatment was investigated in multiple studies [18, 22, 23], and a close connection between the antioxidant and glyoxalase I-II systems during Cd toxicity [24], salt [22] and heat [23] stress was observed. Moreover, since GSH acts as a co-factor in the detoxification of MG, any disturbance of the GSH level and even redox status might have great effects on the overall defense systems of plants exposed to stress conditions. However, previous study also suggested that enhanced Gly II activity will efficiently recycled GSH into the system [21], which facilitated GSH homeostasis and higher antioxidative enzyme activities in preventing Cd stress [25]. Therefore, the balance between endogenous GSH production and glyoxalase system is essential for the MG detoxification and stress tolerance. Previous study has reported that MG pretreatment could trigger the heat tolerance of maize seedlings by driving the AsA-GSH cycle and ROS−/MG-scavenging system [23]. Nevertheless, it is unclear how exogenous MG influences Cd tolerance by regulating GSH metabolism and glyoxalase system.

Hydrogen sulfide (H_2_S), similar to MG, has previously considered as a toxic metabolite, disrupting cell signaling processes, especially those involving ROS [26]. However, it is now known to exhibit multiple positive effects on plants exposed to stress conditions at low concentrations [27–29]. Previous studies have shown that H_2_S is able to regulate seed germination and development, as well as the response to environmental stress [30–32]. Besides its developmental and regulatory roles, exogenous H_2_S can also regulate GSH metabolism as H_2_S is an intermediate product of sulfur in plants, which is a precursor of cysteine synthesizing GSH [32], effectively maintains redox status of plants and alleviates oxidative stress induced by mental toxicity [33, 34]. For example, Alsahli et al. [19] reported that exogenous H_2_S can effectively restore GSH and maintain GSH redox potential, leading to reduced oxidative damage in arsenic (As)-stressed pea plants. Additionally, H_2_S will also coordinates the GSH metabolism and glyoxalase systems to mitigate Cd-induced ROS and MG toxicity in plants [25]. In *Brassica rapa* seedlings, previous study has supported the hypothesis that H_2_S participate in GSH-centered MG tolerance in Cd-stressed plants [31]. However, the relationship between H_2_S-induced Cd stress tolerance and MG in *S. matsudana* seedlings is waiting for answering.

Among the kinds of species of willow, *Salix matsudana* Koidz. is native and one of the most widely distributed and commonly cultivated species of willow in China, and is considered a deciduous fast-growing tree [35]. Moreover, a few clones of *S. matsudana* show high heavy metal tolerance [36]. However, the effects of exogenous H_2_S and MG on Cd tolerance in *S. matsudana* through regulating GSH metabolism and glyoxalase system have not been adequately investigated in previous studies. Clarifying these effects will provide more information on the mechanism of GSH metabolism and glyoxalase system in alleviating Cd-induced oxidative stress. Here, *S. matsudana* cuttings were treated with sodium hydrosulfide (NaHS, a donor of H_2_S) and MG under Cd stress, and the concentrations of Cd in plant tissues, ROS levels, antioxidation-related enzyme activities, the relative expression of stress-related genes, and the redox status of AsA and GSH were determined. The objectives of this study were to: (1) investigate the effects of exogenous NaHS and MG on reducing oxidative stress induced by Cd toxicity; (2) explore the key factor involved in regulating the exogenous NaHS and MG-induced Cd stress tolerance in *S. matsudana*.

## Results

### Effects of exogenous MG and NaHS on plant growth and concentration of cadmium

Cadmium stress led to significant (*p* < 0.05) reductions in the relative plant height and leaf area by 30.47 and 36.50%, respectively, compared with control (Table 1). Contrarily, Cd + MG, Cd + NaHS and Cd + MG + NaHS increased the relative plant height and leaf area by 46.89, 40.09, 53.68 and 39.37, 40.94, 42.52%, respectively, with respect to Cd-stressed plants (Table 1). The seedlings exposed to Cd treatment exhibited the highest Cd concentrations in the root and leaf, while Cd + MG, Cd + NaHS and Cd + MG + NaHS reduced Cd concentrations in root and leaf by 16.50, 36.93, 27.95 and 26.21, 32.59, 52.38% with reference to the Cd-stressed pants (Table 2).

**Table 1:** The relative increase of plant height and leaf area after 40 days of cultivation grown under different treatments

Treatment	Plant high (cm)	Leaf area (cm^2^)
Control	12.70 ± 0.72 a	2.00 ± 0.10 a
Cd	8.83 ± 0.35 b	1.27 ± 0.17 b
MG	13.37 ± 0.12 a	1.97 ± 0.12 a
Cd + MG	12.97 ± 0.65 a	1.77 ± 0.13 a
NaHS	13.70 ± 0.40 a	1.82 ± 0.12 a
Cd + NaHS	13.37 ± 0.69 a	1.79 ± 0.04 a
MG + NaHS	14.17 ± 0.49 a	1.98 ± 0.16 a
Cd + MG + NaHS	12.57 ± 0.72 a	1.81 ± 0.06 a

Values are presented as the mean ± SE (*n* = 3) of three biological replicates. Different letters in each row followed by values indicate significant difference (*p* < 0.05) among treatments

**Table 2:** The concentration of cadmium in *S. matsudana* leaves, stems and roots after 40 days of cultivation grown under different treatments

Treatment	Root (mg kg^− 1^ DW)	Stem (mg kg^− 1^ DW)	Leaf (mg kg^− 1^ DW)
Control	nd	nd	nd
Cd	846.33 ± 47.24 a	197.04 ± 26.19 a	480.50 ± 95.09 a
MG	nd	nd	nd
Cd + MG	706.67 ± 47.31 b	238.63 ± 11.96 a	354.58 ± 21.02 b
NaHS	nd	nd	nd
Cd + NaHS	533.75 ± 57.63 c	152.21 ± 4.47 b	323.92 ± 40.88 bc
MG + NaHS	nd	nd	nd
Cd + MG + NaHS	609.79 ± 33.28 bc	234.42 ± 7.08 a	228.83 ± 17.36 d

Values are presented as the mean ± SE (*n* = 3) of three biological replicates. Different letters in each row followed by values indicate significant difference (*p* < 0.05) among treatments

### Effects of exogenous MG and NaHS on ROS levels and lipid peroxidation

**Fig. 1:**
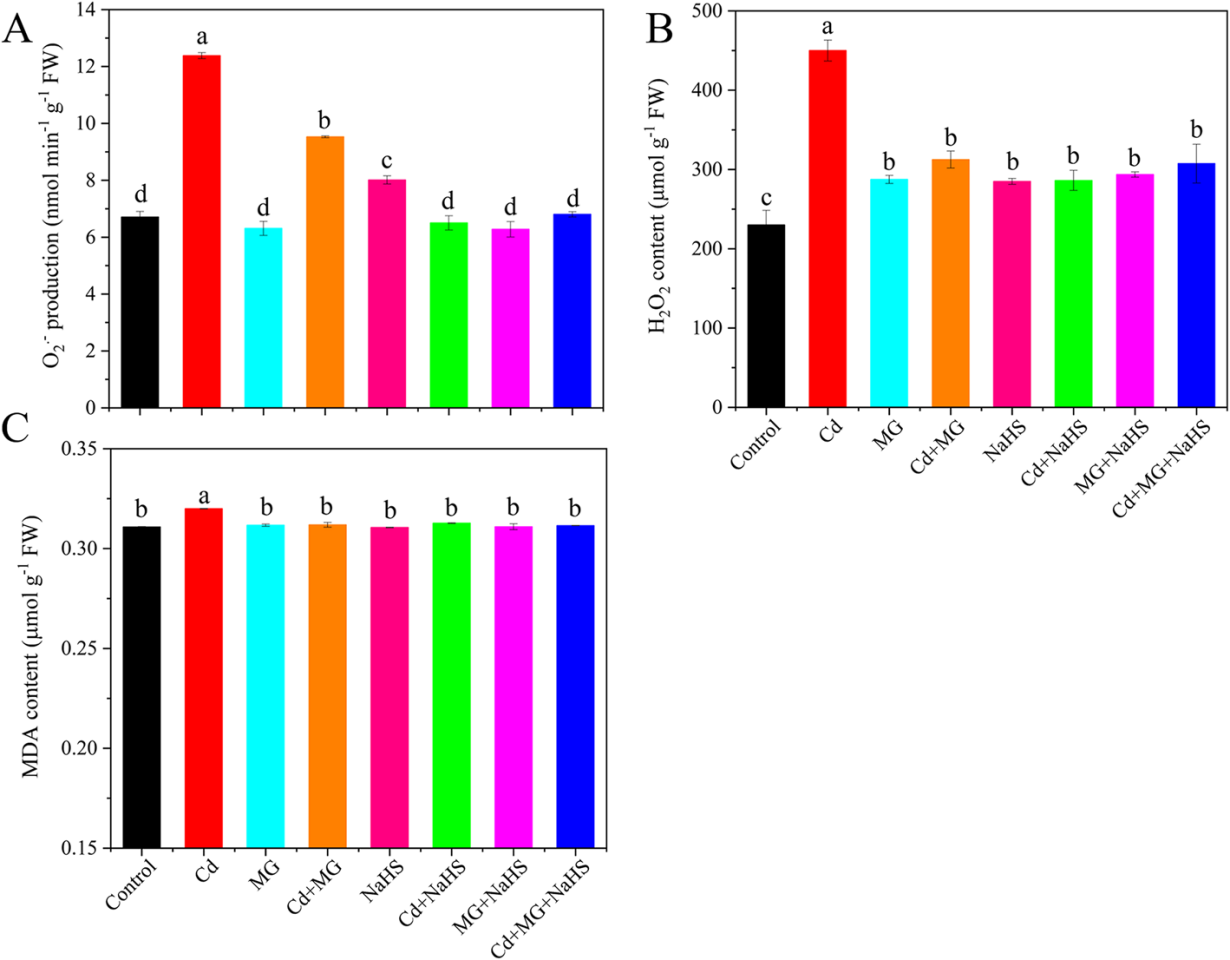
The O_2_^.-^ production rate (**A**), H_2_O_2_ (**B**) and MDA (**C**) content in *S. matsudana* leaves after 40 days of cultivation grown under different treatments. Values are presented as the mean ± SE (*n* = 3) of three biological replicates. Lower case letters above columns indicate significant difference (*p* < 0.05) among treatments

Exposure to Cd promoted O_2_^.-^ production rate and H_2_O_2_ content by 84.46 and 95.65%, respectively, and significantly increased MDA with respect to the control (Fig. 1); however, Cd + MG, Cd + NaHS and Cd + MG + NaHS reduced the O_2_^.-^ production rate, and H_2_O_2_ content by 23.01, 47.47, 45.04 and 30.56, 36.39, 31.67%, respectively, over those in the Cd-stressed plants (Fig. 1A and B). Moreover, the applications of MG and NaHS combined with Cd showed lower (*p* < 0.05) MDA contents relative to Cd treatment (Fig. 1C).

### Effects of exogenous MG and NaHS on antioxidative enzymes and relative expression levels of stress-related genes

**Fig. 2:**
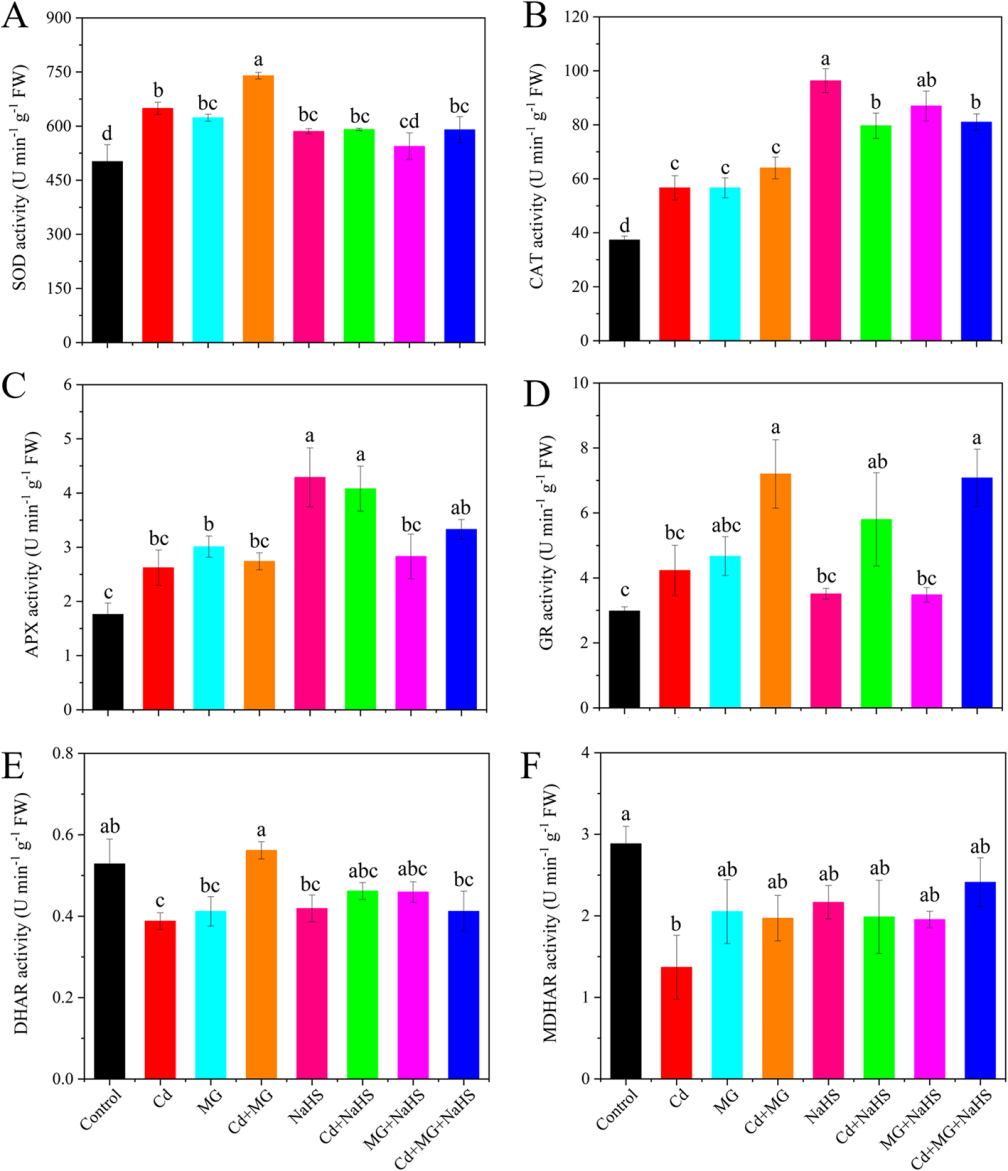
The activities of SOD (**A**), CAT (**B**), APX (**C**), GR (**D**), DHAR (**E**) and MDHAR (**F**) in *S. matsudana* leaves after 40 days of cultivation grown under different treatments. Values are presented as the mean ± SE (*n* = 3) of three biological replicates. Lower case letters above columns indicate significant difference (*p* < 0.05) among treatments

Compared with control, Cd treatment significantly (*p* < 0.05) increased the SOD and CAT activities in *S. matsudana* leaves by 29.41 and 51.79%, respectively (Fig. 2A and B). Moreover, Cd + MG further increased (*p* < 0.05) the SOD and GR activities by 13.94 and 70.42%, respectively, and Cd + NaHS further increased (*p* < 0.05) the CAT and APX activities by 40.59 and 55.68%, respectively, with reference to those in only Cd-treated plants (Fig. 2A, B, C and D). Contrarily, exposure to Cd decreased DHAR and MDHAR activities by 26.58 and 52.54%, respectively, relative to the control, while MG and NaHS application reduced the inhibitory effect of Cd stress (Fig. 2E and F).

**Fig. 3:**
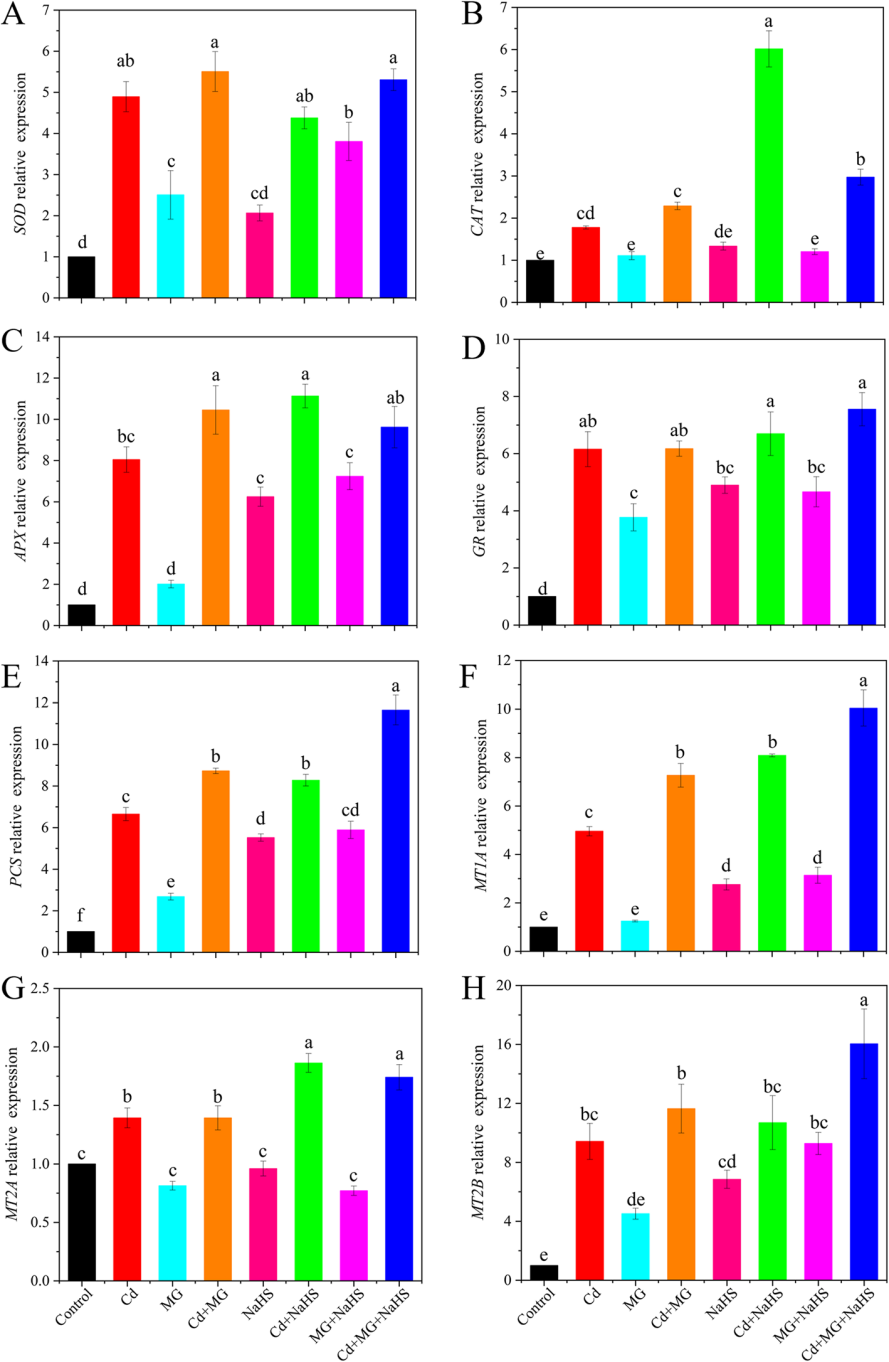
The relative gene expression of *SOD* (**A**), *CAT* (**B**), *APX* (**C**), *GR* (**D**), *PCS* (**E**), *MT1A* (**F**), *MT2A* (**G**) and *MT2B* (**H**) in *S. matsudana* leaves after 40 days of cultivation grown under different treatments. Values are presented as the mean ± SE (*n* = 3) of three biological replicates. Lower case letters above columns indicate significant difference (*p* < 0.05) among treatments

Compared with control, Cd addition significantly (*p* < 0.05) increased the relative expression levels of *SOD*, *CAT*, *APX*, *GR*, *PCS*, Metallothioneins (*MT1A*), *MT2A* and *MT2B* genes (Fig. 3). The highest relative expression of the *CAT* gene was observed under Cd + NaHS treatment, followed by Cd + MG + NaHS, Cd + MG, and Cd treatments (Fig. 3B). Moreover, the relative expression of the *APX* gene under Cd + MG and Cd + NaHS were significantly improved by 29.89 and 38.32%, respectively, in respect of those in Cd-seedlings (Fig. 3C). Furthermore, Cd + MG, Cd + NaHS and Cd + MG + NaHS increased the relative expression levels of *PCS* and *MT1A* genes by 31.28, 24.51, 75.26 and 46.67, 63.20, 102.44%, respectively, with reference to those in the plants grown under Cd stress alone (Fig. 3E and F). In addition, Cd + NaHS and Cd + MG + NaHS improved the relative expression levels of *MT2A* and *MT2B* genes by 33.64, 24.91 and 13.46, 70.24%, respectively, compared with Cd-stressed plants (Fig. 3G and H).

**Fig. 4:**
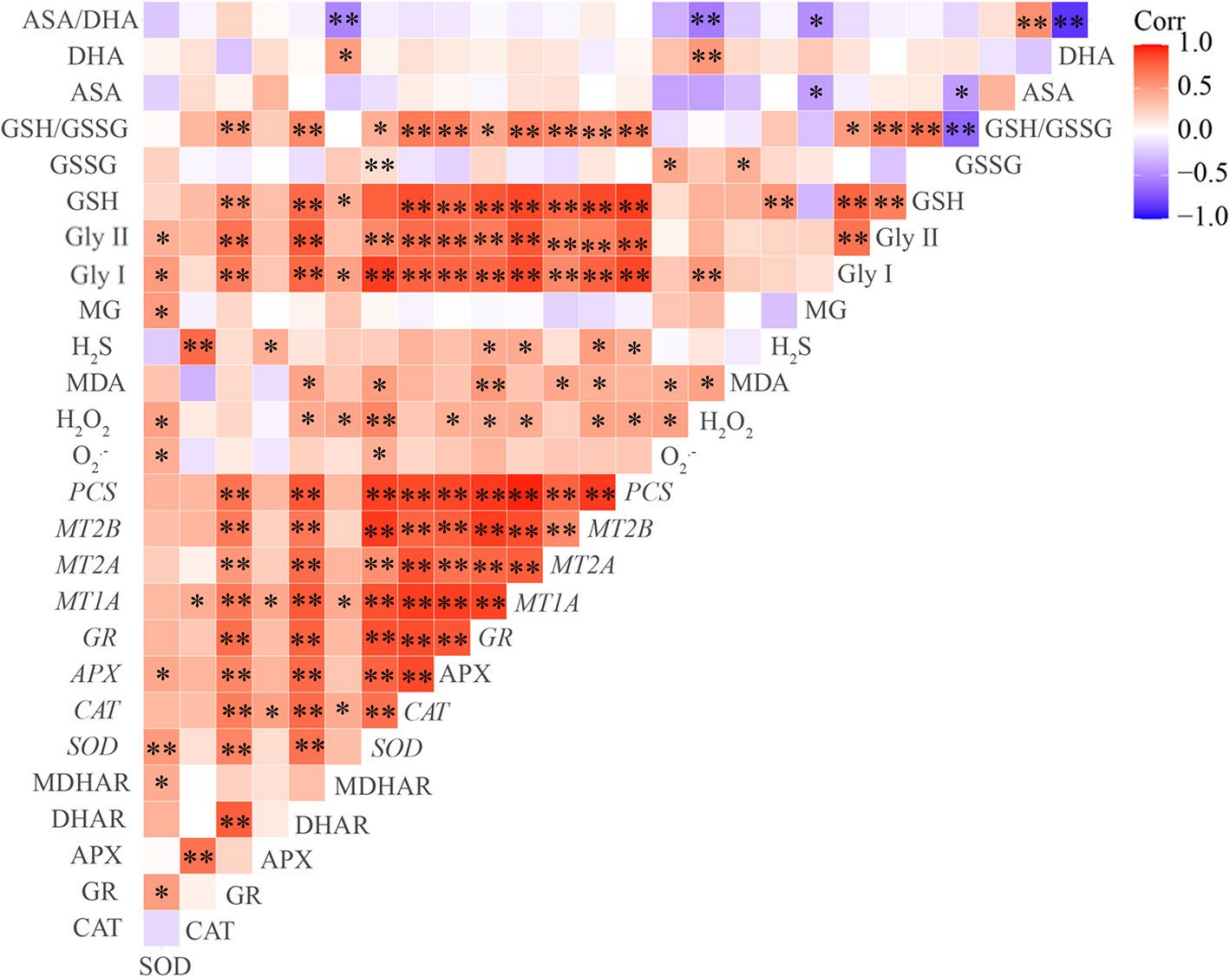
The correlations between the ROS level, lipid peroxidation, antioxidative enzyme activities, relative expression of stress-related genes, H_2_S and MG contents were determined by Spearman test. * indicates significant correlation at 0.05 level; ** indicates significant correlation at 0.01 level

Spearman correlation analysis showed that the H_2_O_2_ content and O_2_^.-^ production rate was positively (*p* < 0.05) correlated with SOD activity (Fig. 4). The relative expression levels of *SOD* and *GR* genes were positively (*p* < 0.01) related to SOD and GR activities, respectively (Fig. 4). The H_2_S content was positively (*p* < 0.05) linked to the relative expression levels of *GR*, *MT1A*, *MT2B*, and *PCS* genes (Fig. 4).

### Effects of exogenous MG and NaHS on non-enzymatic antioxidants, MG, and H_2_S contents and glyoxalase system enzymes

**Fig. 5:**
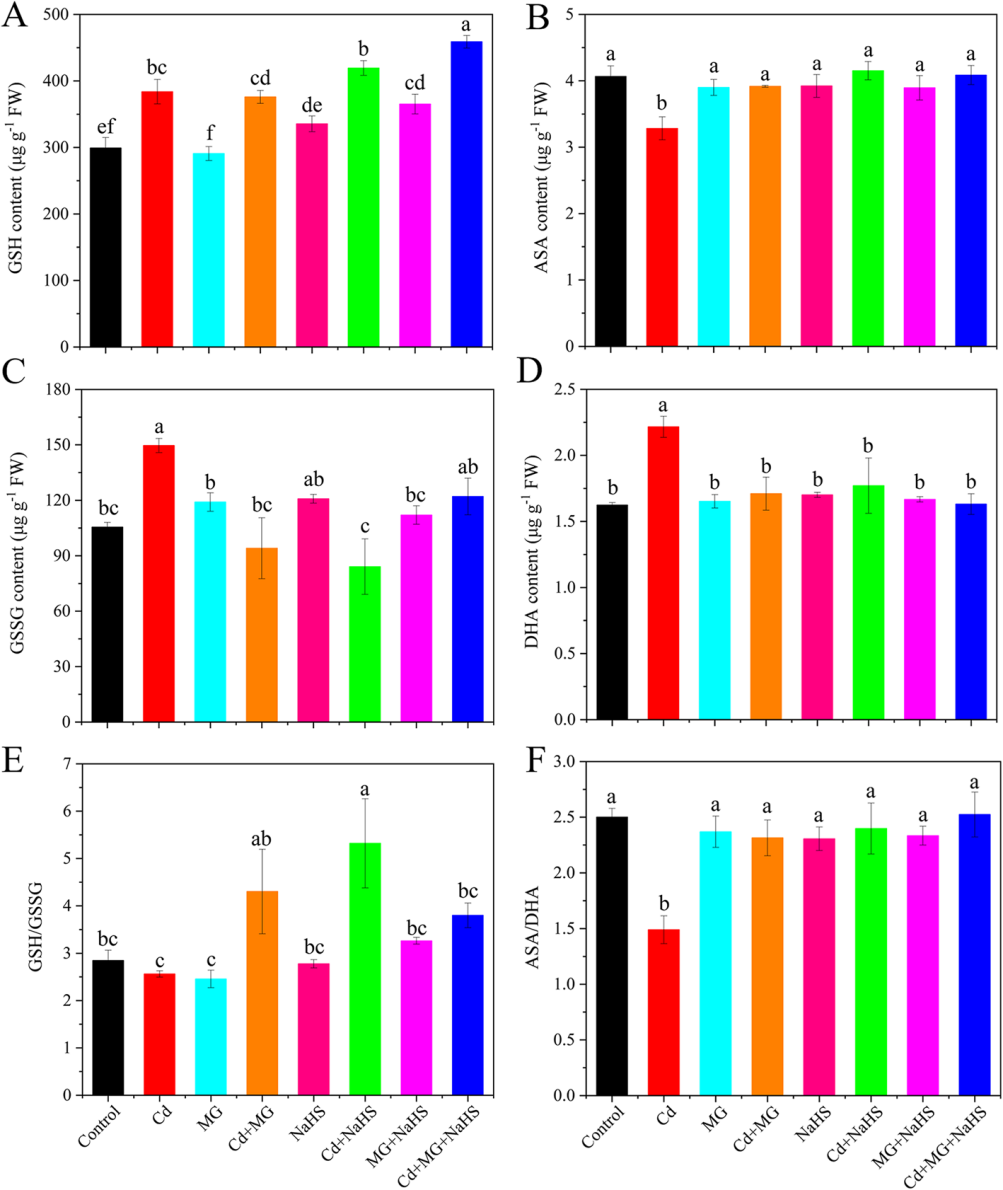
The contents of GSH (**A**), GSSG (**C**), AsA (**B**), and DHA (**D**) on fresh weight (FW) basis and the ratios of GSH/GSSG (**E**) and AsA/DHA (**F**) in *S. matsudana* leaves after 40 days of cultivation grown under different treatments. Values are presented as the mean ± SE (*n* = 3) of three biological replicates. Lower case letters above columns indicate significant difference (*p* < 0.05) among treatments

Compared with control, exposure to Cd increased the contents of GSH, GSSG, and DHA by 28.24, 41.80, and 36.40%, respectively, but decreased the AsA content and the ratios of GSH/GSSG and AsA/DHA by 19.23, 9.97, and 40.44%, respectively (Fig. 5). In contrast, Cd + MG, Cd + NaHS and Cd + MG + NaHS increased the ratios of GSH/GSSG and AsA/DHA by 67.96, 107.72, 48.33 and 55.34, 60.99, 69.40%, respectively, compared to those in the Cd-treated plants (Fig. 5).

**Fig. 6:**
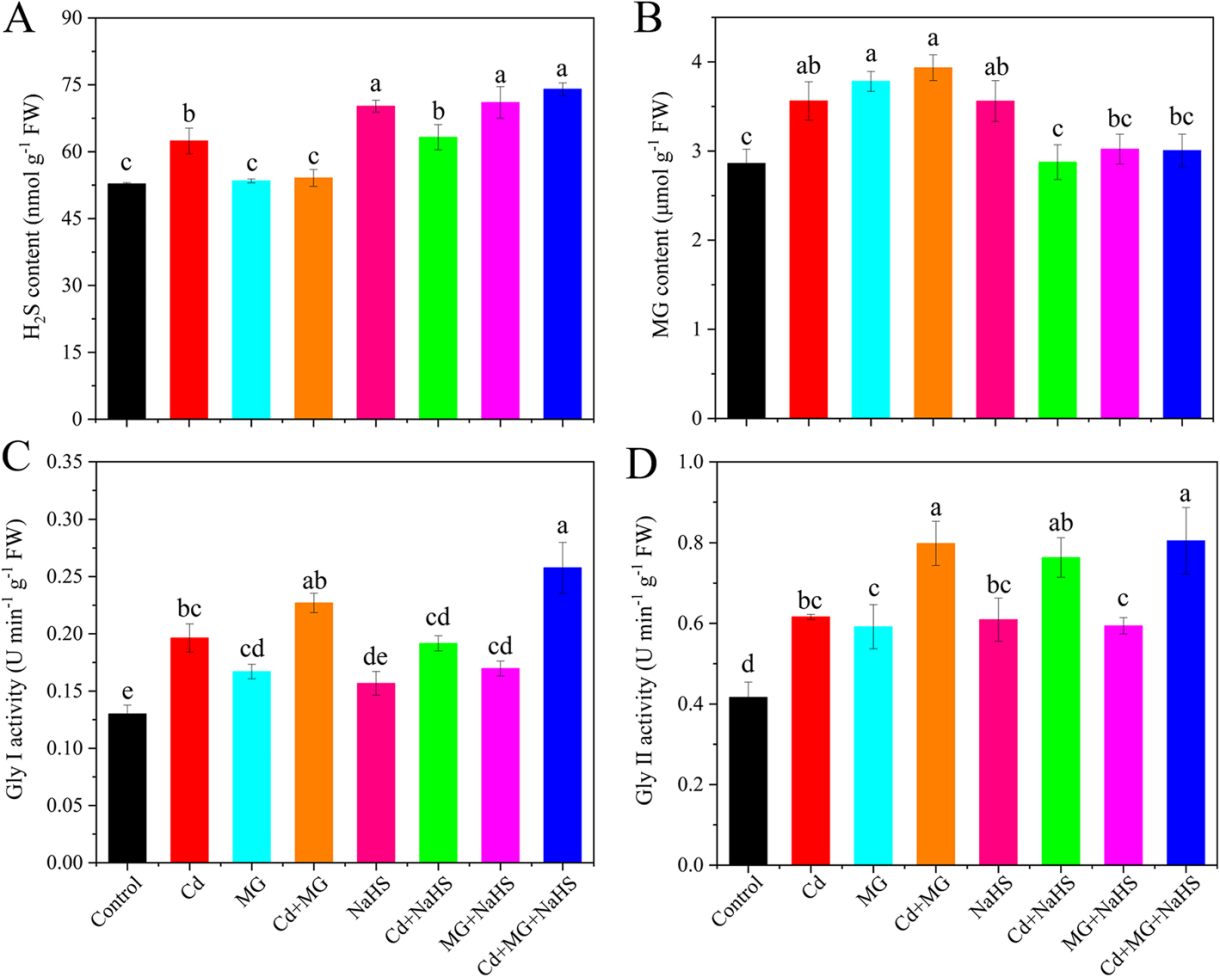
The contents of H_2_S (**A**) and MG (**B**) and the activities of Gly I (**C**) and Gly II (**D**) in *S. matsudana* leaves after 40 days of cultivation grown under different treatments. Values are presented as the mean ± SE (*n* = 3) of three biological replicates. Lower case letters above columns indicate significant difference (*p* < 0.05) among treatments

Relative to control, exposure to Cd increased the H_2_S and MG contents by 18.22 and 24.51%, respectively (Fig. 6A and B). Moreover, NaHS, MG + NaHS, and Cd + MG + NaHS treatments further increased the H_2_S contents (Fig. 6A), while MG and Cd + MG further improved the MG contents compared with Cd stress alone (Fig. 6B). Application of Cd markedly increased the Gly I and Gly II activities by 51.03 and 47.99%, respectively, compared with control (Fig. 6C and D). In addition, the Gly I and Gly II activities were further enhanced under Cd + MG and Cd + MG + NaHS treatments with respect to Cd-stressed plants (Fig. 6C and D).

The results of Spearman correlation showed that the GSH content and the ratio of GSH/GSSG were positively (*p* < 0.05) related to the H_2_S content, Gly I, Gly II, GR, and DHAR activities as well as the relative expression levels of stress-related genes (Fig. S1). The DHA content and the ratio of AsA/DHA were markedly (*p* < 0.01) linked to the H_2_O_2_ content (Fig. S1).

### Relationships among physiological traits of treatments

**Fig. 7:**
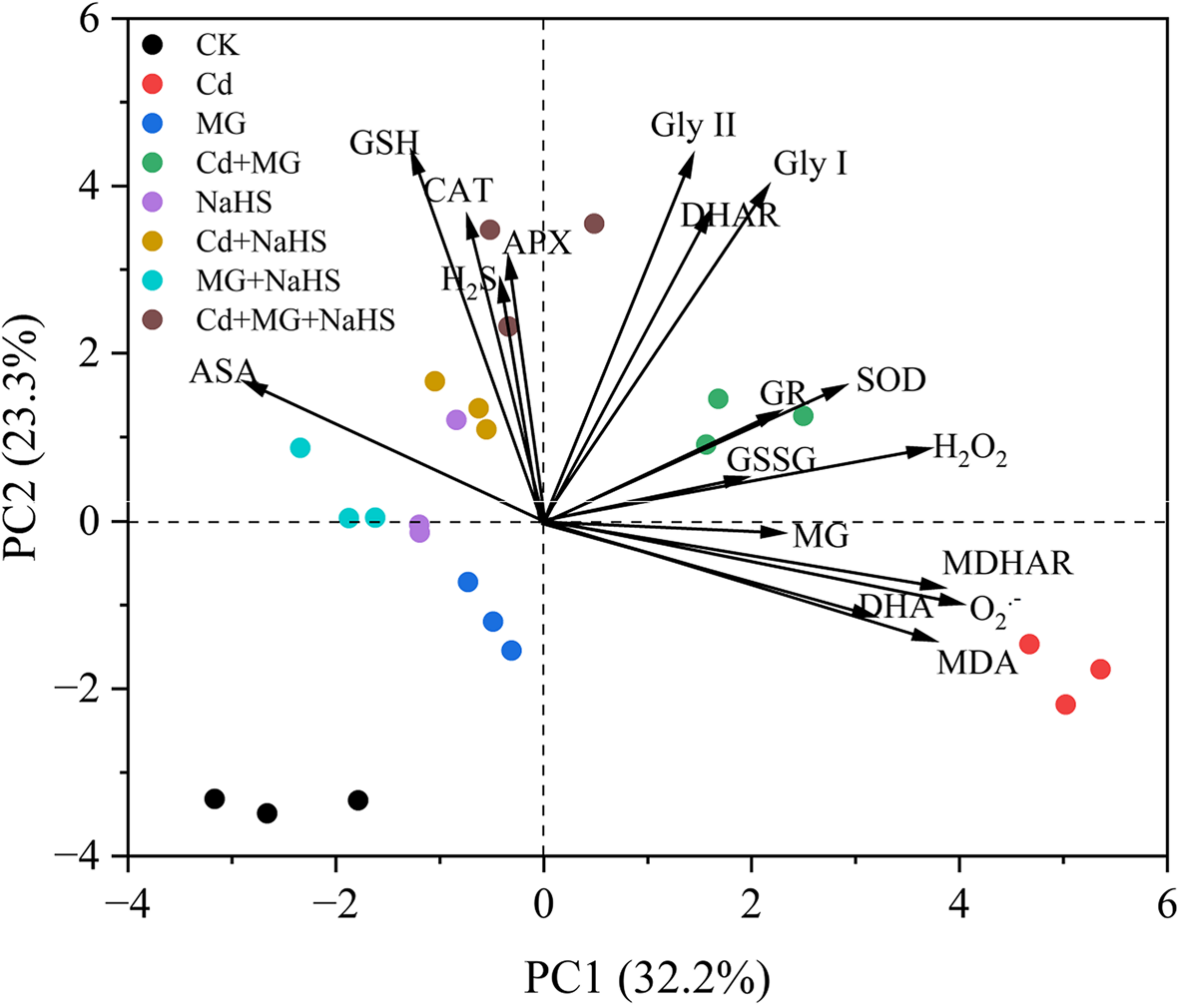
Principal component analysis (PCA) based on eco-physiological traits in *S. matsudana* among treatments. The biplot graph showing PCA score and loadings of different studied attributes of the leaves of Cd stressed willow seedling supplemented with NaHS and MG

The biplot (score and loading) resulting from the PCA evaluated the effects of exogenous applications of MG and NaHS on physiological and biochemical attributes of *S. matsudana* exposed to Cd stress (Fig. 7). The PC1 (32.2%) and PC2 (23.3%) showed maximum contribution to the total variance in the given database. All 8 treatments were obviously dispersed by the first two principal components. The PCA results revealed that MDA, O_2_^.-^, and MDHAR were significant contributors in PC1 and were strongly related to Cd stress, while SOD and H_2_O_2_ were strongly related to Cd + MG treatment. Moreover, GSH, CAT, H_2_S, and APX were closely linked to Cd + MG + NaHS treatment.

### Contributions of non-enzymatic antioxidant components, antioxidative enzymes, and glyoxalase system to plant growth

**Fig. 8:**
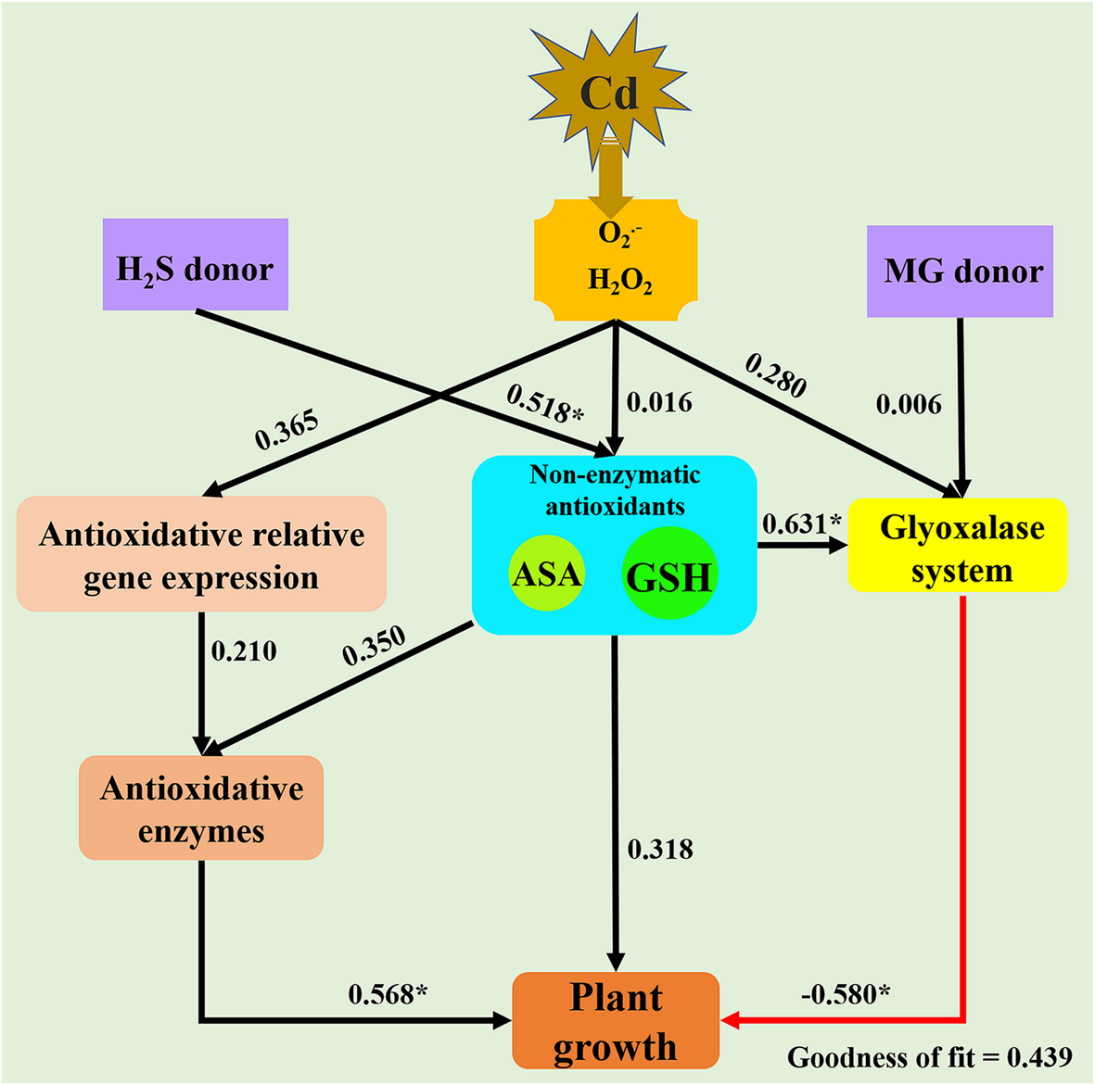
Partial least squares path models (PLS-PM) of the drivers of *S. matsudana* growth. Non-enzymatic antioxidants included the contents of GSH, GSSG, ASA, DHA and the ratios of GSH/GSSG and ASA/DHA; glyoxalase system included Gly I and Gly II activities; antioxidative relative genes included *SOD*, *CAT*; *APX*, *GR*; antioxidative enzymes included SOD, CAT; APX, GR; plant growth is represented by the relative plant heigh. Each oblong box represents a latent variable. Path coefficients were calculated after 1000 bootstraps. The black and red lines represent positive and negative effects, respectively. * indicates significant contribution at 0.05 level

The PLS-PM was constructed to reveal the relative contributions of antioxidative enzymes, non-enzymatic antioxidants, and glyoxalase system to the improvement of plant growth (Fig. 8). The application of NaHS exhibited stronger contribution to non-enzymatic antioxidants (path coefficient = 0.518) than the contribution of MG addition to glyoxalase system (path coefficient = 0.006). The ROS levels (H_2_O_2_ content and O_2_^.-^ production rate) showed stronger positive influence on the antioxidation-related gene expression (path coefficient = 0.365) than the glyoxalase system (path coefficient = 0.280). Furthermore, non-enzymatic antioxidants exhibited higher contribution to the glyoxalase system (path coefficient = 0.631) than antioxidative enzymes (path coefficient = 0.350). The antioxidative enzymes (path coefficient = 0.568) showed highest contribution to plant growth, followed by non-enzymatic antioxidants (path coefficient = 0.318).

## Discussion

In plants, Cd toxicity is generally indicated as stunted growth of the root and shoot, chlorosis, and inhibition of photosynthesis [37, 38]. In the present study, Cd stress sharply inhibited *S. matsudana* growth (Tables 1), which may have occurred due to the perturbation in cellular processes at the biochemical and physiological levels in plants [19, 39]. However, exogenous applications of MG and NaHS restored these growth parameters (Table 1). Similarly, exogenous supplementation of MG and NaHS has also been reported to improve the plant growth parameters under chromium [29] and Cd toxicity [39]. The mitigated effect of NaHS and MG on growth inhibition may be due to the less accumulation of Cd in the aboveground and its translocation from the root to shoot [29], evidenced as the significant reduction of Cd concentrations in the root and leaf compared with Cd treatment alone (Table 2). Our results were in line with previous study showing that exogenous NaHS reduced Cd uptake in *Brassica rapa* seedlings [31].

Exposure to Cd damages the cell membranes and disturbs the mechanism of photosynthesis, which result in overaccumulation of ROS beyond the plant scavenging capacity [40]. In this study, plants exposed to Cd significantly developed more oxidative stress compared with control (Fig. 1). These results were in accordance with the previous studies on rice seedlings [41] and Arabidopsis plant [42]. In contrast, supplementation of NaHS and MG to the Cd-stressed plants reduced ROS levels and MDA content, indicating an essential function of NaHS and MG in alleviating oxidative damages. This may be result from the induction of the antioxidant defense system by H_2_S and MG, which act as the signal molecules [19, 43].

In response to oxidative stress, plants are capable of upregulating the relative expression of stress-related genes and enhancing the activities of various antioxidative enzymes to detoxify ROS in plant cells [4, 38]. In this study, the activities of SOD, CAT, APX, and GR and their corresponding relative gene expression levels were stimulated by Cd stress (Figs. 2 and 3). These activating responses may be related to the Cd toxicity tolerance strategy, as reported in previous studies [2, 5]. Moreover, exogenous applications of MG and NaHS further elevated the stimulation effects (Figs. 2 and 3). In line with our results, the application of H_2_S was reported to alleviate oxidative damages in *Medicago sativa* by boosting the activities of antioxidant enzymes and the quantity of their respective transcripts [39]; thus, indicating the important role of antioxidative enzymes in ROS detoxification. In addition to the stimulation of antioxidative enzymes and relative gene expression, NaHS was also reported to enhance the antioxidant capacity by inactivating NADPH oxidase (which is responsible for the ROS production) [32]; thus, reducing the overproduction of ROS. Moreover, exogenous supplementation of NaHS and MG maintained the redox status of AsA and GSH through regulating the AsA–GSH cycle enzymes (Figs. 2 and 5) [26], thereby triggering the tolerance of plants to Cd [12].

Another major self-defense strategy of heavy metal stressed plants is the compartmentalization of metal ions in the vacuoles, which is common in woody plants [35]. The functional groups of GSH or its derivatives, such as MTs and PCs, can sequester the complexes to reduce the cellular metal toxicity by binding Cd ions with S-containing amino acid ligands [35]. A previous study illustrated that MTs and PCs were induced by heavy metal stress and were modulated at the transcriptional level [15]. Therefore, the up-regulated gene expression levels of *PCS*, *MT1A*, *MT2A* and *MT2B* under MG and NaHS applications (Fig. 3) suggested the contributions of MTs and PCs to the metal tolerance through chelating metal ions and improving the homeostasis of metals [44].

The results of this study revealed high contribution of non-enzymatic antioxidants to antioxidative enzymes (Fig. 8), indicating the important role of GSH and AsA in scavenging ROS [45]. Furthermore, the GSH/GSSG and AsA/DHA ratios indicate the intracellular redox potential and is important in ROS regulation [17]. In this study, exposure to Cd resulted in reduced ratios of GSH/GSSG and AsA/DHA compared with the control (Figs. 5E and F), demonstrating that Cd stress caused disturbance in the redox status of the cell by disrupting AsA and GSH pools. However, NaHS and MG applications restored the redox status, as shown by higher ratios of GSH/GSSG and AsA/DHA (Figs. 5E and F), indicating stronger stress tolerance characteristics [10]. The increasement of GR, DHAR and Gly II activities under Cd + NaHS and Cd + MG (Figs. 2D, E and 6D) may responsible for the higher GSH/GSSG and AsA/DHA ratios, as GR catalyses the reduction of GSSG to GSH and DHAR is responsible for AsA regeneration [46], and Gly II is able to recycled GSH into the system [21] to maintain the GSH level. Therefore, the enhanced ratios of GSH/GSSG and AsA/DHA correspond with the improved activities of GR, DHAR and Gly II suggested the crucial roles of NaHS and MG in triggering the up-regulation of the non-enzymatic antioxidants defense system. Similarly, previous studies have also reported that the addition of NaHS and MG was able to ameliorate Cd toxicity stress by re-establishment of the redox status [31, 33].

In plants, MG can function as both a cytotoxin at high concentration and signal molecule at low concentration [18]. In the present study, Cd, MG and Cd + MG treatments all significantly increased MG content in the leaves of *S. matsudana*, compared with control, and MG and Cd + MG treatments showed higher MG contents than Cd-stressed plants (Fig. 6B). These results were consistent with Wang et al. (2019) [47] and Li et al. (2018) [30] who reported that maize treated with MG under heat stress showed higher MG content than heat stressed seedlings without MG pretreated, but also had higher survival percentage. However, the inconsistent results have also been observed by Li et al. (2018) [33] who found that combined applications of Cd and MG markedly reduced MG content in wheat compared with Cd-stressed alone. These contradictory results are likely to be explained by differences in plant species and MG concentrations as well as the experimental methods (pot experiment vs hydroponics). However, the seedlings treated with MG and Cd + MG showed better growth potential than Cd stressed plants (Table 1). Therefore, the inhibition effect on growth parameters of *S. matsudana* is likely resulted from the oxidative damage caused by Cd-induced overproduction of ROS (Fig. 1). In addition, the Cd- and MG-induced ROS production under MG and Cd + MG treatments may be eliminated faster by the antioxidant enzymes and AsA-GSH cycle than Cd stressed plants, which was evidenced by promoted antioxidant defence system (Figs. 2 and 5) and significant lower O_2_^.-^ production rate, H_2_O_2_ and MDA content (Fig. 1). Furthermore, plants have developed a unique glyoxalase system, including Gly I and Gly II, which precisely regulates MG homeostasis via synergistic effect with GSH [22]. The rise in Gly I and Gly II activities induced by Cd stress in the present study (Fig. 6C and D) has also been reported on rice seedlings under salt stress [48] and *Brassica juncea* under Zn toxicity [49], indicating that Cd-induced MG might act as a signal to enhance the capacity of MG detoxification [43]. In addition, Cd + MG showed higher Gly I and Gly II activities than Cd-stressed alone (Fig. 6C and D), which was similar with previous study [33], indicating that enhanced glyoxalase system may be able to maintain MG within a certain range which is unable to disrupt the normal functioning of cells and metabolic behavior in *S. matsudana* seedlings [33]. However, the efficiency of MG detoxification by the glyoxalase I-II system strongly relies on the GSH level in plants [50, 51], evidenced as the significant contribution of non-enzymatic antioxidants to the glyoxalase system (Fig. 8) as well as the positive links between GSH content and glyoxalase system (Fig. S1). GSH participated in the MG detoxification by acting as a substrate in the glyoxalase system [52], and the increased endogenous GSH level has been shown to alleviate MG toxicity and oxidative stress under various abiotic stresses as it stimulated the antioxidant and glyoxalase system [10, 13, 14]. In the present study, exogenous applications of NaHS and MG maintained higher Gly II activity and stable endogenous GSH level in Cd-affected seedlings compared with Cd addition alone (Figs. 5D and 6E), which was in line with the studies on pea [19], wheat [33] and rice seedlings [29]. Thus, the enhanced glyoxalase enzyme activities and GSH level induced by exogenous NaHS and MG strongly contribute to MG detoxification.

In the present study, the redox status of AsA and GSH showed strong contributions to the glyoxalase system and antioxidative enzymes, which finally affected the plant growth directly or indirectly (Fig. 8). These results revealed the important role of GSH and AsA in reducing the oxidative stress and maintaining the cellular redox potential under abiotic stress tolerance [11, 16]. Previous study on mung bean also confirmed the important role of GSH and AsA on antioxidant defense system and MG detoxification [10]. However, the results of the present study highlighted that GSH exhibited more sensitive responses to the exogenous applications of NaHS and MG compared with AsA (Fig. 5), and it seemed to play a more important role in alleviating oxidative stress through influencing antioxidative enzymes and glyoxalase system (Fig. S1). This may be attributed to the following mechanisms: Firstly, exogenous applications of NaHS and MG stimulated GR activity and relative expression of *GR* (Figs. 2D and 3D), therefore improved GSH metabolism, leading to higher capacity of ROS detoxification [27]. Secondly, GSH participated in MG detoxification by acting as a co-factor in the glyoxalase system, and it was considered as a limiting factor in MG detoxification [18]. Therefore, the multivariate functions of GSH determined a more active regenerative system of GSH than that of AsA. We suggested that GSH is the key factor in modulating exogenous NaHS and MG-induced Cd stress tolerance in *S. matsudana*, and the scavenging of ROS by AsA may depend on the level of GSH.

## Conclusions

**Fig. 9:**
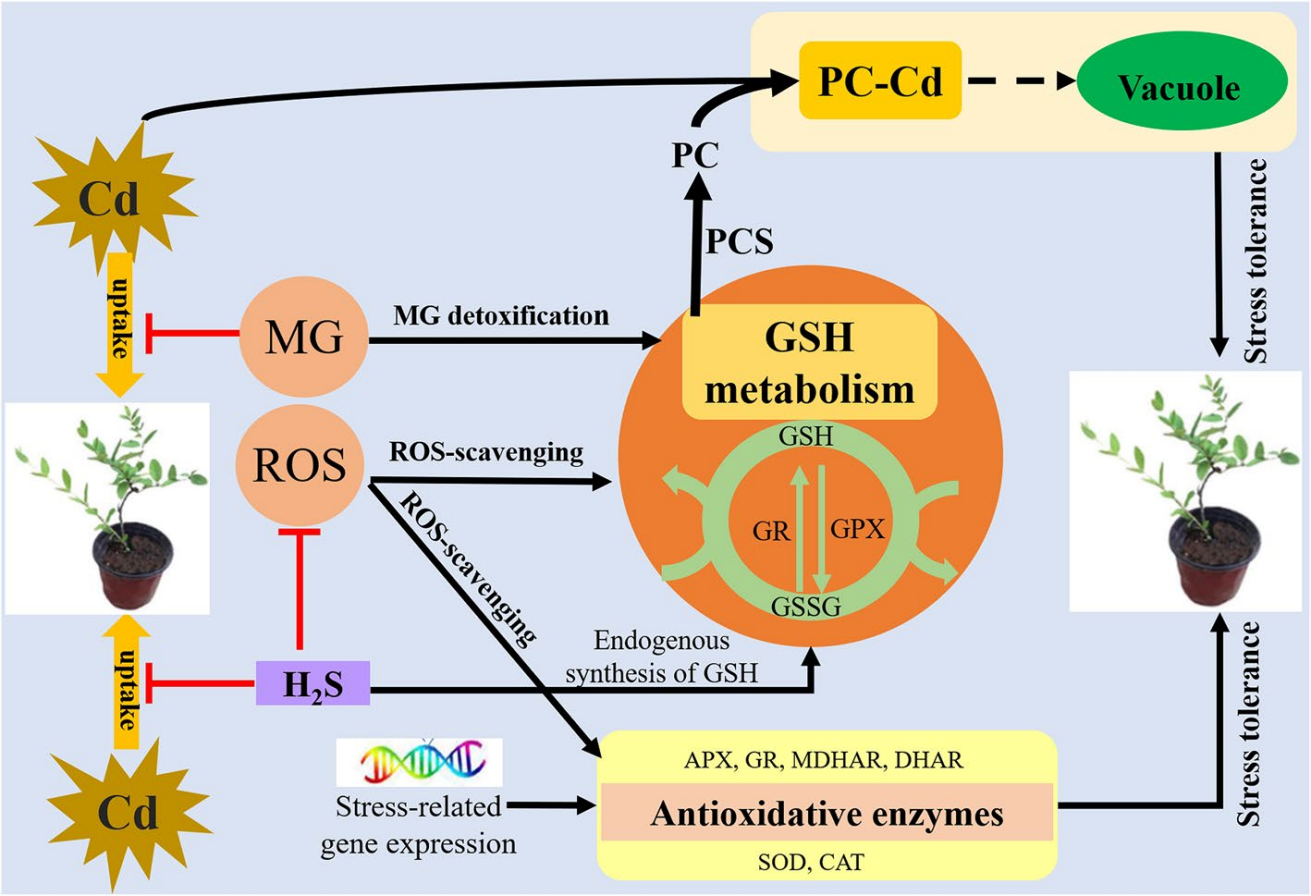
The underlying mechanisms of GSH metabolism in alleviating exogenous NaHS and MG-induced Cd stress tolerance in *S. matsudana*. The blunted arrows (┬) denote inhibitory effects

In conclusion, the present study indicated that exogenous applications of NaHS and MG reduced the oxidative stress and restored the growth parameters in Cd-treated *S. matsudana* seedlings through reducing the Cd uptake and increasing the antioxidative enzyme activities and relative expression levels of stress-related genes. Furthermore, exogenous applications of NaHS and MG accelerated the GSH metabolism through increasing the enzymes of the AsA-GSH cycle and glyoxalase system, therefore maintained redox status of AsA and GSH, resulting in decreased oxidative stress in plants (Fig. 9). Moreover, compared with AsA, GSH plays a more important role in regulating the exogenous NaHS and MG-induced Cd stress tolerance in *S. matsudana*. Considering the crucial multivariate role of GSH in Cd stress tolerance induced by exogenous NaHS and MG, further studies is essential to reveal the mechanism of exogenous GSH in enhancing metal stress tolerance under Cd stress condition.

## Methods

### Pot experiment and plant sampling

The brown topsoil (pH 7.06) used in the experiment was air-dried and filtered through a 4.0 mm sieve. Similar sized stem cuttings (15 ~ 20 cm in height) were collected during September, 2016 from branches of the same adult mother plants, one clone of *S. matsudana* grown on the campus of Liaoning University, Shenyang, China. Then, the stem cuttings were cultivated on the sandy soil in the greenhouse on September 26, 2016, and then they were transplanted to the soil on October 11, 2016.

Cadmium chloride (CdCl_2_·2.5H_2_O) and sodium hydrosulfide (NaHS) were used as Cd and H_2_S donors, respectively. Eight treatments were prepared as follows: (1) Control; (2) Cd (150 mg kg^− 1^); (3) MG (108 mg kg^− 1^); (4) Cd + MG; (5) NaHS (50 mg kg^− 1^); (6) Cd + NaHS; (7) MG + NaHS; (8) Cd + MG + NaHS. The soil and reagents were thoroughly mixed according to the treatments on April 11, 2017, and then they were preincubated for 30 days before planting. Each treatment had three replicate pots with dimensions of 30 cm (height) × 29 cm (open top) × 21 cm (flat bottom), and four *S. matsudana* cuttings with a similar growth and height were directly planted in each pot on May 11, 2017. Therefore, each treatment contains twelve *S. matsudana* seedlings in total (three biological replicate pot × four seedlings in each pot). Soil moisture was maintained at 60% field capacity by adding deionized water once a day. All pots were randomly placed in natural environment in Liaoning University (Shenyang, Northeast China). This region has continental monsoon climate, with an annual average temperature and precipitation of 7.9 °C and 705 mm, respectively. Leaf samples from the same part (middle part) of the seedlings in each pot were collected at the 40th day after planting. Leaf samples within one pot were pooled into a single composite sample, wrapped in tin foil, immediately frozen in liquid nitrogen, and stored at − 80 °C in the laboratory until subsequent analysis.

### Determination of growth parameters and cadmium concentration

The relative plant height (cm) and leaf area (A, cm^2^) of *S. matsudana* cuttings of each treatment were measured after 40 days growth. Additionally, individual *S. matsudana* samples were divided into roots, leaves, and stems after harvest. All samples were thoroughly washed three times with deionized water, oven-dried at 80 °C until constant weight was achieved. The dried samples (0.5 g of each sample) were milled to flour with a stainless steel grinder and digested with the double acid (HNO_3_–HClO_4_, 4:1) method [53]. The concentrations of Cd in each sample were determined using an atomic absorption spectrometer (Analyst 400, PerkinElmer, CT, USA).

### Determination of lipid peroxidation and ROS levels

Lipid peroxidation was indicated by the content of malondialdehyde (MDA), which was measured using thiobarbituric acid (TBA) [40]. Briefly, 0.1 g leaves were extracted with 10 mL of 10% trichloroacetic acid (TCA) and then centrifuged at 10,000×g for 20 min. The equal volume of TBA was added to the supernatant and incubated at 95 °C for 30 min, and then it was cooled on ice immediately. The absorbances at 450, 532, and 600 nm were measured using an ultraviolet spectrophotometer (UV-2100, UNICO, Shanghai, China) after centrifugation 20 min at 10,000×g.

The hydroxylamine hydrochloride (HONH_3_Cl) method with minor modifications was used to determine the rate of O_2_^.-^ generation in leaves [1]. Briefly, 0.1 g leaves were ground in 3 mL 0.05 mol L^− 1^ phosphate buffer (pH 7.8), and then they were centrifuged at 5000 rpm for 3 min at 4 °C. Subsequently, the phosphate buffer (pH 7.8) and 1 mol L^− 1^ HONH_3_Cl were added to the 0.5 mL supernatant and incubated for 20 min at 25 °C. Then, 17 mmol L^− 1^ p-aminobenzene sulfonic acid and 7 mmol L^− 1^ 1-naphthylamine were mixed with the solution and measured at 530 nm.

The hydrogen peroxide (H_2_O_2_) content was determined as the method proposed by Patterson et al. [54]. In brief, 0.5 g leaves were ground in 2.5 mL propanone and then centrifuged at 12,000 rpm (4 °C for 10 min). The mixture containing 0.1 mL 5% titanium sulphate and 0.2 mL ammonia was added to the resulting supernatant and centrifuged at 10,000 rpm at 4 °C for 10 min. The resulting precipitate was re-centrifuged after dissolving using 2 mol L^− 1^ H_2_SO_4_. The H_2_O_2_ content was determined at 415 nm.

### Determination of antioxidant enzymes and non-enzymatic antioxidants

The superoxide dismutase (SOD; EC 1.15.1.1) activity in leaves was determined by the rate of inhibition of nitroblue tetrazolium (NBT) reduction at 560 nm, with xanthine oxidase as a H_2_O_2_ producing agent, as previously described [55]. The amount of enzyme required for 50% inhibition of the photochemical reduction of NBT was defined as one unit of SOD activity.

Catalase (CAT; EC 1.11.1.6) activity was measured by the method proposed by Bashir et al. [2]. In brief, the total volume of 3.0 mL reaction solution comprising 1.5 mL of 50 mM of phosphate buffer (pH 7.0), 0.2 mL enzyme extract, 1 mL of 1 M H_2_O_2_, and 0.3 mL distilled water was used to test the CAT activity by calculating the reduction in absorbance measured at 240 nm within 3 min. One unit of CAT was determined by the amount decomposing 1 μmol of H_2_O_2_ within 1 min.

The enzymes of the AsA-GSH cycle were determined as previously described [56]. In brief, fresh leaves (0.5 g) were extracted using 3.5 mL 50 mmol L^− 1^ phosphate buffer (pH 7.8) containing 1.0 mmol L^− 1^ EDTA-Na_2_, 2% (v/v) polyvinylpyrrolidone (PVP), 1.0 mmol L^− 1^ ascorbate, and 1.5 mL saturated ammonium sulfate. The mixture was used to determine the following enzyme activities after centrifugation at 5000 rpm (4 °C for 10 min):

Ascorbate peroxidase (APX; EC 1.11.1.11) activity: A total volume of 1 mL reaction mixture containing phosphate buffer (pH 7.0), 0.83 mL AsA, 0.13 mL H_2_O_2_, and 0.04 mL crude enzyme was utilized. The reduction in absorbance measured at 290 nm over 1 min was considered as the AsA consumption. The extinction coefficient of 2.8 mmol L^− 1^ cm was used to calculate APX activity.

Dehydroascorbate reductase (DHAR; EC 1.8.5.1) activity: A reaction mixture (1 mL) containing 0.7 mL phosphate buffer, 0.1 mL dehydroascorbate (DHA), 0.1 mL reduced GSH, and 0.1 mL crude enzyme solution was utilized to determine the DHAR activity. DHAR activity was calculated from the differences in absorbance at 265 nm within 1 min, with the extinction coefficient of 14 mmol L^− 1^ cm.

Monodehydroascorbate reductase (MDHAR; EC 1.6.5.4) activity: The reaction mixture containing phosphate buffer (pH 7.6), 0.03 mL NADPH, 0.9 mL AsA, 0.04 mL ascorbate oxidase, and 0.03 mL of crude enzyme solution was utilized. The consumption of NADPH was used to determine the MDHAR activity, which was tested by monitoring the changes in absorbance at 340 nm within 1 min. The extinction coefficient of 6.2 mmol L^− 1^ cm was employed to calculate the MDHAR activity.

Glutathione reductase (GR; EC 1.6.4.2) activity: The reaction mixture containing 0.1 mL NADPH, 0.86 mL oxidized glutathione (GSSG), and 0.04 mL of crude enzyme extract was utilized. GR activity was determined by the reduction in absorbance at 340 nm within 1 min, using an extinction coefficient of 2.8 mmol L^− 1^ cm.

For the determination of non-enzymatic antioxidants, fresh leaves (0.5 g) were extracted using 3 mL of ice-cold 5% meta-phosphoric acid containing 1 mM EDTA, and then the extracted solution was centrifuged at 11,500×g for 15 min. Reduced and total AsA contents were measured at 265 nm in 100 mM K–P buffer (pH 7.0) with 1.0 U of ascorbate oxidase according to the method proposed by Dutilleul et al. [57]. The DHA content was calculated by deducting the reduced AsA amount from the total AsA content. The total GSH and GSSG contents were determined by the method proposed by Griffith [58] based on enzymatic recycling. Reduced GSH content was calculated by deducting the amount of GSSG from the total GSH content.

### Determination of H_2_S and MG contents and glyoxalase system enzymes

The methos of Christou et al. [59] with modifications was used to determine the H_2_S content in *S. matsudana* leaves. Briefly, 0.25 g leaves were extracted using 1 mL of 100 mM K–P buffer (pH 7.0) containing 10 mM EDTA and centrifuged at 11,200×g for 15 min. After centrifugation, 1880 μL extraction buffer and 20 μL of 20 mM DTNB [5,5′-dithiobis (2-nitrobenzoic acid)] were added to the homogenized supernatant (100 μL) and incubated at 25 °C for 5 min. The H_2_S content was determined at 412 nm.

The MG content was measured by the method of Yadav et al. [18]. Specifically, MG was extracted by 1 mL of 0.5 M perchloric acid (HClO_4_) in 0.1 g of leaves. The extraction was incubated on ice for 15 min and centrifuged at 13,000 rpm for 20 min at 4 °C. The supernatant was neutralized with saturated potassium carbonate and incubated for 15 min at room temperature and then centrifuged at 13,000 rpm for 10 min. The neutralized supernatant was used for the estimation of MG. In a total volume of 1 mL, 250 μL of 7.2 mM 1,2-diaminobenzene, 100 μL of 5 M HClO_4_, and 650 μL of the neutralized supernatant were added in that order. The MG concentration was determined at 335 nm. The concentration of MG was expressed as μmol g^− 1^ fresh weight (FW) (μmol g^− 1^ FW).

Glyoxalase I (Gly I) and Gly II activities were determined according to the method proposed by Hasanuzzaman et al. [60]. A mixed solution of 100 mM K–P buffer (pH 7.0) containing 1.7 mM GSH, 15 mM magnesium sulphate, and 3.5 mM MG was added to the enzyme assay solution. The Gly I activity was assessed at 240 nm. For Gly II activity, a mixed solution of 100 mM Tris−HCl buffer at pH 7.2 containing 1 mM S-D-lactoylglutathione and 0.2 mM DTNB was added to the enzyme solution. Finally, the Gly II activity was recorded at 240 nm.

### Total RNA extraction and real-time quantitative PCR analysis

The total RNA was extracted from 0.5 g frozen *S. matsudana* leaf tissues using Trizol reagent (Invitrogen, USA) following the manufacturer’s protocol, and the quantity was measured with the Nano Drop UV-VIS 2000 spectrophotometer (Thermo Fisher Scientific, Waltham, MA, USA). About 2 μg of total RNA was used for the first strand cDNA synthesis using a cDNA synthesis kit (Takara, Dalian, China).

The expression levels of stress-related genes were analyzed using quantitative PCR (Q-PCR) on an ABI 7500 Real-Time PCR System (Applied Biosystems, Germany). Gene-specific primers of each gene for Q-PCR are listed in Table S1. The 20 μL reaction system was prepared with SYBR Green Real-Time PCR Master Mix (Takara, Dalian, China). Three biological replicates of each sample were performed and the expression levels were determined using the 2^-∆∆Ct^ method by standardizing the cycle threshold (Ct) value for each gene relative to the Ct value of ACTIN. The data are presented as the fold change in gene expression normalized to an endogenous reference gene and relative to the untreated control [61].

### Statistical analysis

The values of each sample were processed using SPSS 20.0 (SPSS Inc., Chicago, IL, USA), based on one-way analysis of variance (ANOVA) with Duncan’s tests (*p* < 0.05). Origin (version 2022) was used for graphical representations. The correlations between the ROS levels, MDA content, antioxidative enzyme activities, relative expression levels of stress-related genes, H_2_S and MG contents, Gly I and Gly II activities, and non-enzymatic antioxidants were determined by Spearman test in SPSS 20.0 (SPSS Inc., Chicago, IL, USA). In order to depict the relationships among different observations and response variables, the principal component analysis (PCA) was performed and visualized in Origin (version 2022). Partial least squares path modeling (PLS-PM) was conducted using the “plspm” package in R (version 4.0.2) to reveal the effects of non-enzymatic antioxidants, antioxidative enzymes, and glyoxalase system on plant growth, which was indicated by the relative plant height.

## Supplementary Information

**Additional file 1: Table S1.** Gene-specific primers sequences used in the present study. **Fig. S1.** The correlations between the ROS levels, MDA content, antioxidative enzyme activities, stress-relative gene expressions, H_2_S and MG contents, Gly I and Gly II activities and non-enzymatic antioxidants were determined by Spearman test. * indicates significant correlation at 0.05 level; ** indicates significant correlation at 0.01 level.

## Data Availability

All study data are included in the manuscript and its additional files.

## Abbreviations

APX: ascorbate peroxidase
AsA: ascorbate
Cd: cadmium
CAT: catalase
DHA: dehydroascorbate
DHAR: dehydroascorbate reductase
Gly I: glyoxalase I
Gly II: glyoxalase II
GR: glutathione reductase
GSH: reduced glutathione
GSSG: oxidized glutathione
H_2_O_2_: hydrogen peroxide
H_2_S: hydrogen sulfide
MDA: malondialdehyde
MDHAR: monodehydroascorbate reductase
MG: methylglyoxal
MT: metallothionein
NaHS: sodium hydrosulfide
O_2_^.-^: superoxide radical
PCS: phytochelatin synthase
ROS: reactive oxygen species
SOD: superoxide dismutase
