# Supplementary material for: Exogenous hydrogen sulfide and methylglyoxal alleviate cadmium-induced oxidative stress in Salix matsudana Koidz by regulating glutathione metabolism
Source: BMC Plant Biol. 2023 Feb 2;23:73. doi: 10.1186/s12870-023-04089-y (PMC9893619; doi:10.1186/s12870-023-04089-y)
Supplement: Supplementary file 1 — Additional file 1: Table S1. Gene-specific primers sequences used in the present study. Fig. S1. The correlations between the ROS levels, MDA content, antioxidative enzyme activities, stress-relative gene expressions, H2S and MG contents, Gly I and Gly II activities and non-enzymatic antioxidants were determined by Spearman test. * indicates significant correlation at 0.05 level; ** indicates significant correlation at 0.01 level. [file 12870_2023_4089_MOESM1_ESM.docx]

Supplementary Material

**Table S1** Gene-specific primers sequences used in the present study.

| Gene | Primer Sequence (5′-3′) | Accession number |
| --- | --- | --- |
| *SOD* | F: AGGTGTCTGTGGCACCATCT | KM591686.1 |
|  | R: AAATGCGGCCCAGTTGACAT |  |
| *CAT* | F: CTGCCCTGCTATTGTGGTTCCTGGT | KM591687.1 |
|  | R: AATCGTTCTTGCCTGTCTGGTGCCC |  |
| *APX* | F: CAGCACCAGCAACGGAAATA | KM591688.1 |
|  | R: TCGCCAACCGAATAAAGAGA |  |
| *GR* | F: TCTCCTCCCGCCGTCCTCTCTATCA | KM591685.1 |
|  | R: GCCTACGCCTCCAGTTGTCTCCGAA |  |
| *MT1A* | F: AGAATGGCTGCAAGTGCGGATC | EF157299.1 |
|  | R: ACACATGGACTGACCACCTGATTG |  |
| *MT2A* | F: AAACTCTTGTTCTTGGTGTG | EF157297.1 |
|  | R: AAGTGATTCTTCTCTGGTGC |  |
| *MT2B* | F: AGAACCCCAGAAATGAGACA | EF157299.1 |
|  | R: GAAACACCTTGAAAGTTGAAGCAGC |  |
| *PCS* | F: AGTGTGTCTGGCTCATTGTG | HQ228353.1 |
|  | R: GTGCTTTGATTTGTTCGGTA |  |


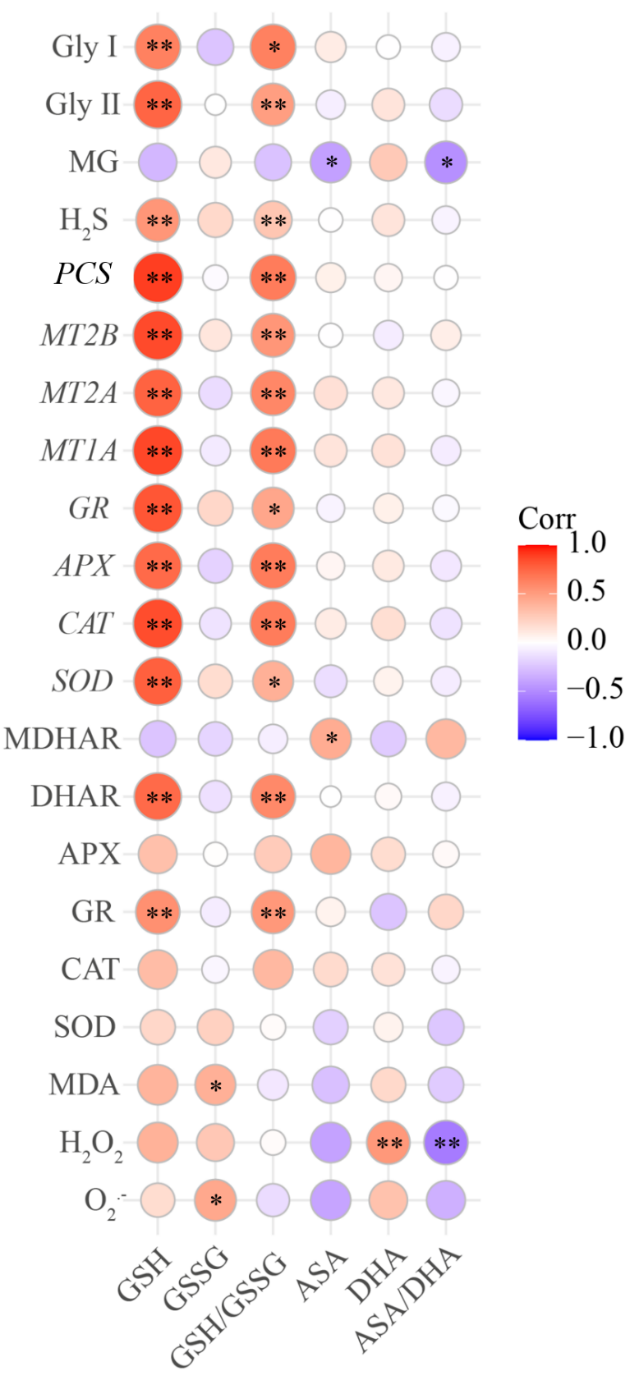


**Figure S1.** The correlations between the ROS levels, MDA content, antioxidative enzyme activities, stress-relative gene expressions, H_2_S and MG contents, Gly Ⅰ and Gly Ⅱ activities and non-enzymatic antioxidants were determined by Spearman test. * indicates significant correlation at 0.05 level; ** indicates significant correlation at 0.01 level.
